# Transcatheter Aortic Valve Replacement Reverses Heyde Syndrome: A Case Report of Severe Aortic Stenosis and Gastrointestinal Bleeding

**DOI:** 10.3390/jcm14082819

**Published:** 2025-04-19

**Authors:** Claudiu Florin Rășinar, Alexandru Tîrziu, Rebeca Ionela Rășinar, Florin Gîru, Cristian Mornoș, Dan Gaiță, Constantin Tudor Luca, Daniel Miron Brie

**Affiliations:** 1 Cardiovascular Disease Institute Timisoara, Gheorghe Adam St., No. 13A, 300310 Timisoara, Romania; claudiu.rasinar@umft.ro (C.F.R.); florin.giru@umft.ro (F.G.); mornos.cristian@umft.ro (C.M.);; 2 Doctoral School, “Victor Babes” University of Medicine and Pharmacy Timisoara, Eftimie Murgu Square, No. 2, 300041 Timisoara, Romania; 3 Department of Functional Sciences, “Victor Babes” University of Medicine and Pharmacy, Tudor Vladimirescu Street, No. 14, 300174 Timisoara, Romania; 4 Gastroenterology Clinic, Clinical Emergency County Hospital Timișoara, Liviu Rebreanu St., No. 156, 300723 Timisoara, Romania; 5 Research Center of the Institute of Cardiovascular Diseases, Cardiovascular Disease Institute Timisoara, Gheorghe Adam St., No. 13A, 300310 Timisoara, Romania; 6 Department of Cardiology, “Victor Babes” University of Medicine and Pharmacy Timisoara, Eftimie Murgu Square, No. 2, 300041 Timisoara, Romania

**Keywords:** Heyde syndrome, aortic stenosis, transcatheter aortic valve replacement (TAVR), gastrointestinal bleeding, angiodysplasia, von Willebrand factor (vWF), acquired von Willebrand syndrome, video capsule endoscopy (VCE)

## Abstract

**Background:** Heyde syndrome is a rare condition characterized by the triad of severe aortic stenosis, gastrointestinal bleeding, and acquired type 2A von Willebrand syndrome. This case report highlights the diagnostic and therapeutic approach for a 72-year-old woman presenting with exertional chest pain, dyspnea, fatigue, and a history of melena. **Methods:** The diagnostic workup revealed severe microcytic anemia and a reduced vWF ristocetin-to-antigen ratio. Imaging confirmed severe degenerative aortic stenosis, while video capsule endoscopy identified angiodysplasia and telangiectasias in the small bowel as the source of gastrointestinal bleeding. Following evaluation by a multidisciplinary Heart Team, the patient underwent transcatheter aortic valve replacement (TAVR) with an Evolut Fx self-expanding prosthesis. **Results:** Post-procedural echocardiography showed mild paravalvular regurgitation. The patient’s clinical course was favorable, with resolution of anemia and no further gastrointestinal bleeding episodes. **Conclusions:** Heyde syndrome requires a high index of suspicion for diagnosis in patients with severe aortic stenosis and unexplained anemia or gastrointestinal bleeding. TAVR offers an effective treatment option that not only resolves valvular pathology, but also mitigates associated bleeding risks.

## 1. Introduction

The current treatment of symptomatic severe aortic stenosis in the elderly involves transcatheter aortic valve replacement (TAVR) [1]. TAVR has shown remarkable results when compared with conventional surgery in elderly patients (long-term mortality or stroke), even in patients at low surgical risk, and has recently become the preferred method in this category of patients [2]. Many elderly patients have some degree of anemia, with multifactorial causes. More than 10% of patients over the age of 65 meet the World Health Organization (WHO) criteria for defining anemia [3,4]. There is also an increased incidence (40%) of anemia in patients with aortic stenosis [5]. The association between aortic stenosis, gastrointestinal bleeding leading to anemia, and acquired von Willebrand syndrome is known as Heyde syndrome. In 1958, Dr. Heyde described in a letter to the *New England Journal of Medicine* (NEJM) the increased incidence of gastrointestinal bleeding in patients with significant calcification of the aortic valve [6]. The mechanism showing the involvement of the von Willebrand factor was described much later [7]. Currently, studies concerning the epidemiology of Heyde syndrome report a prevalence of 1–3%, with a higher bleeding risk compared to non-Heyde patients (9.8% vs. 21.2%, *p*  =  0.03, one year after TAVI) and a higher need for transfusion therapy (50.0% vs. 31.9%, *p* = 0.03) [8].

We present the case of a 72-year-old woman diagnosed with Heyde syndrome who recovered after the percutaneous implantation of an Evolut Fx self-expanding aortic valve.

## 2. Case Presentation

We report the case of a 72-year-old woman who presented to the emergency department with exertional precordial pain, dyspnea on exertion, and significant fatigue. Her medical history revealed the occurrence of melena two weeks prior. The symptoms began approximately six months previously and had progressively worsened over time. In the past two weeks, her condition had deteriorated further, significantly impacting her daily activities and overall quality of life. As a result, she had progressively reduced her physical activity and gradually withdrawn from social activities.

Her medical history included arterial hypertension diagnosed 15 years ago, for which she was on antihypertensive therapy, maintaining well-controlled blood pressure at home. Additionally, she had been receiving lipid-lowering therapy for dyslipidemia for the past five years. The most recent laboratory tests, conducted two years ago, showed hypercholesterolemia, normal renal and liver function, and no signs of anemia. Her last cardiology consultation was four years ago, at which time the patient was diagnosed with moderate aortic stenosis. She was a non-smoker and did not consume alcohol.

On admission, her clinical status was stable. She had a normostenic body constitution (BMI = 26 kg/m^2^; waist circumference = 78 cm; BSA = 1.78 m^2^) with pale skin and mucous membranes, mild bilateral lower limb edema with pitting on pressure, a blood pressure of 130/80 mmHg (with no significant difference between arms while on antihypertensive treatment), a heart rate of 93 bpm, and an oxygen saturation of 93% on room air. Pulmonary auscultation revealed preserved vesicular breath sounds bilaterally with mild basal crackles. Cardiac auscultation identified regular tachycardic heart sounds and a systolic murmur of maximum intensity at the aortic focus, radiating bilaterally to the carotid arteries.

Laboratory investigations revealed normal renal and liver function, severe microcytic hypochromic anemia with moderate iron deficiency, elevated NT-proBNP levels, normal cardiac enzyme markers, and normal coagulation parameters, including platelet count, prothrombin time (PT), INR, activated partial thromboplastin time (aPTT), and fibrinogen.

The laboratory findings from day 0 to day 7 of admission are summarized in Table 1.

A 12-lead electrocardiogram (ECG) revealed sinus rhythm with repolarization-phase abnormalities suggestive of left ventricular hypertrophy (LVH).

The anteroposterior chest X-ray showed mild central pulmonary congestion without pulmonary consolidation or pleural effusion. Abdominal ultrasound revealed no significant pathological changes.

**Figure 1:**
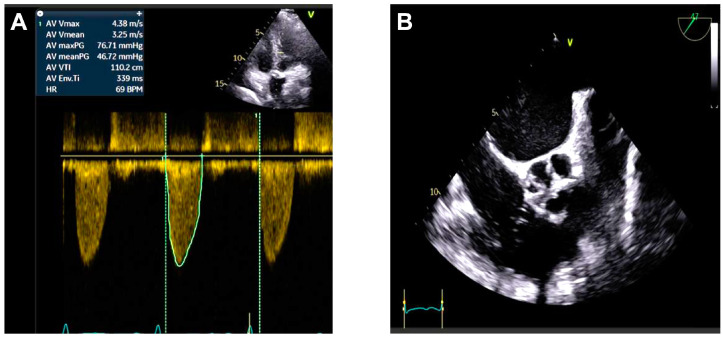
(**A**) Transthoracic echocardiography (TTE) images of the patient: the maximum peak gradient (AV maxPG) across the AV was 76.71 mmHg, the maximum systolic flow velocity (AV Vmax) was 4.38 m/s, and the mean gradient (AV mean PG) was 46.72 mmHg. (**B**) Transesophageal echocardiography showing a severely calcified aortic valve, suggestive of severe aortic stenosis.

**Figure 2:**
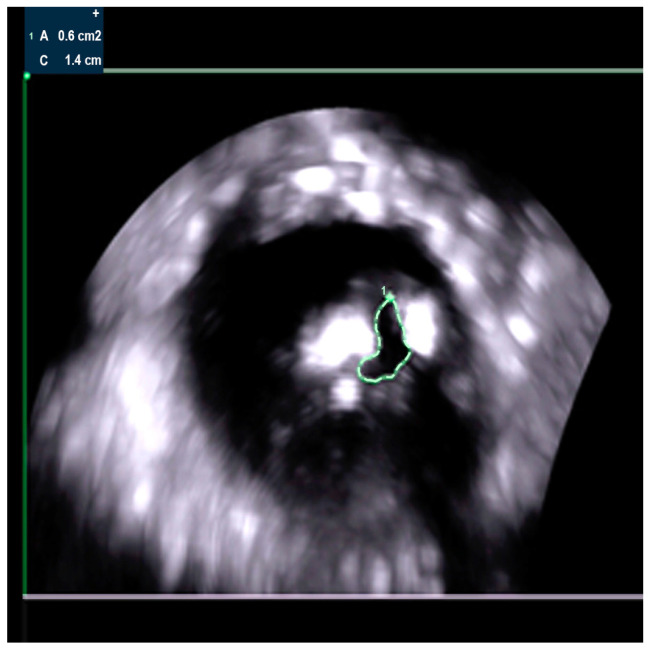
Aortic valve area (AVA) of 0.60 cm^2^, measured by planimetry in TOE (green outline).

**Figure 3:**
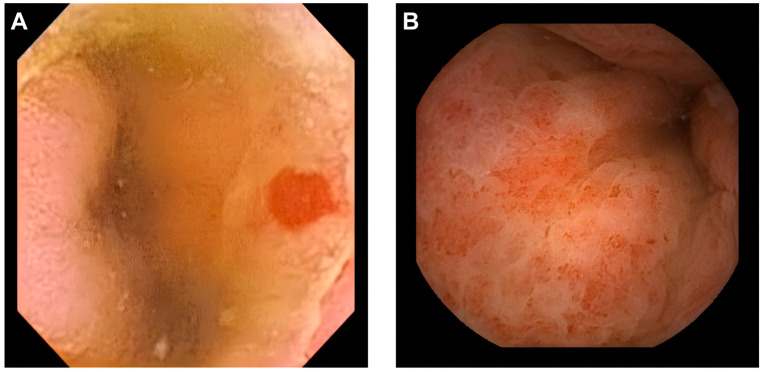
Video capsule endoscopy demonstrating vascular lesions in the small bowel. (**A**) Angiodysplasia is visualized as a discrete, reddish, ectatic lesion on the mucosal surface. (**B**) Telangiectasias appear as multiple, punctate, red spots scattered throughout the small bowel mucosa, indicative of dilated capillaries.

Transthoracic echocardiography (Figure 1) and transesophageal echocardiography (Figure 2 and Figure 3) demonstrated severe degenerative aortic stenosis, with normal global and segmental left ventricular (LV) systolic function, concentric LV hypertrophy, and no pericardial effusion.

On the first day of hospitalization, the patient received two units of red blood cell transfusion (RBC), followed by an additional unit on the second day, leading to the partial correction of anemia. Loop diuretics were administered in intravenous boluses, resulting in progressive decongestion and a favorable clinical response.

A fecal immunochemical test (FIT) was performed, yielding a positive result, prompting a gastroenterology consultation. Gastroscopy and colonoscopy were subsequently performed but did not identify any active bleeding source.

Simultaneously, a hematology consultation was requested, and hematological tests were performed—von Willebrand factor (vWF) antigen, factor VIII (fVIII), vWF ristocetin cofactor activity (vWF/Rco), and the vWF antigen/Rco ratio—leading to the diagnosis of von Willebrand disease type 2A.

Given the diagnosis of Heyde syndrome and the patient’s history of a previous episode of melena, the gastroenterologist requested the administration of a video capsule endoscopy to perform an imaging investigation of the small bowel, which revealed images of angiodysplasia (Figure 3A) and telangiectasias (Figure 3B) in the small bowel.

**Figure 4:**
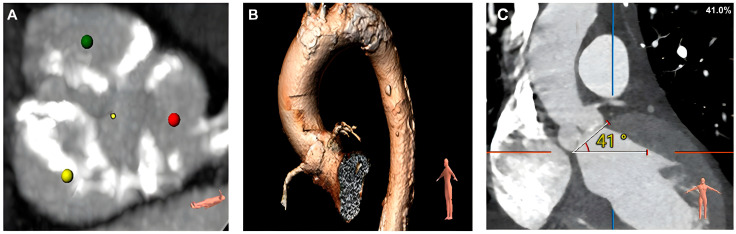
Calcified aortic leaflets (**A**) and mildly dilated ascending thoracic aorta with calcified plaques (**B**). (**A**) Detailed view of calcified aortic valve leaflets, highlighted by color markers (green, yellow, and red) that correspond to different regions of interest. Calcification is visible as dense areas in the images, indicating mineral deposits that reduce leaflet flexibility and affect valve function. (**B**) A three-dimensional reconstruction of the ascending thoracic aorta reveals mild dilation and the presence of calcified plaques along the vessel wall. The calcifications are depicted as dense, irregular structures within the aortic wall. (**C**) Computed tomography angiography demonstrating the angle between the aortic annulus plane and the axial plane (annular angulation) at 41°, suitable for TAVR.

**Figure 5:**
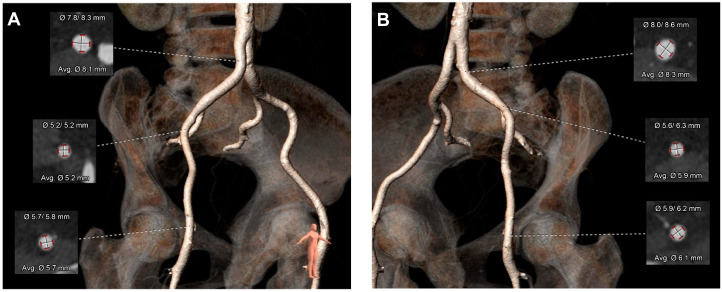
Pre-procedural imaging demonstrating favorable iliofemoral anatomy for transcatheter aortic valve replacement (TAVR). (**A**) Three-dimensional reconstruction of the right iliofemoral artery shows adequate vessel diameter and minimal tortuosity, supporting safe vascular access for TAVR. Cross-sectional measurements at multiple levels indicate average diameters ranging from 5.2 mm to 8.1 mm, ensuring compatibility with delivery systems. (**B**) Corresponding reconstruction of the left iliofemoral artery reveals similarly favorable dimensions, with average diameters ranging from 5.9 mm to 8.3 mm and smooth vessel pathways.

A Heart Team evaluation was conducted, concluding that the patient met the criteria for aortic valve replacement (AVR). To determine eligibility for a transcatheter aortic valve implantation (TAVR) procedure, a thoracic aorta CT angiography (TAVR protocol—Figure 4A–C) and bilateral iliac artery CT angiography (Figure 5A,B) were performed, confirming suitability for the procedure.

**Figure 6:**
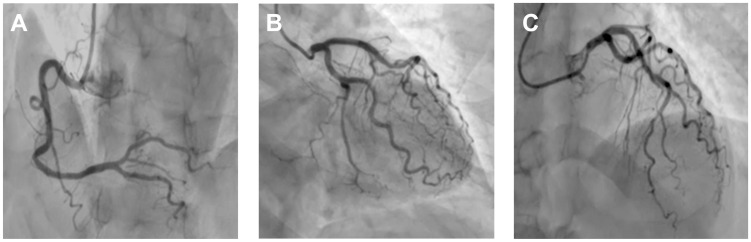
Coronary angiography demonstrating no significant atherosclerotic lesions. (**A**) Right coronary artery visualized in the left anterior oblique (LAO) cranial projection, showing smooth vessel contours and uninterrupted blood flow. (**B**) Left coronary artery depicted in the right anterior oblique (RAO) caudal projection, illustrating clear lumen without evidence of stenosis or irregularities. (**C**) Left coronary artery visualized in the left anterior oblique (LAO) cranial projection, confirming favorable anatomy and absence of obstructive lesions. These findings suggest optimal coronary perfusion conditions and low risk of ischemic complications.

Coronary angiography revealed diffusely atheromatous epicardial coronary arteries without significant angiographic stenosis (Figure 6).

**Figure 7:**
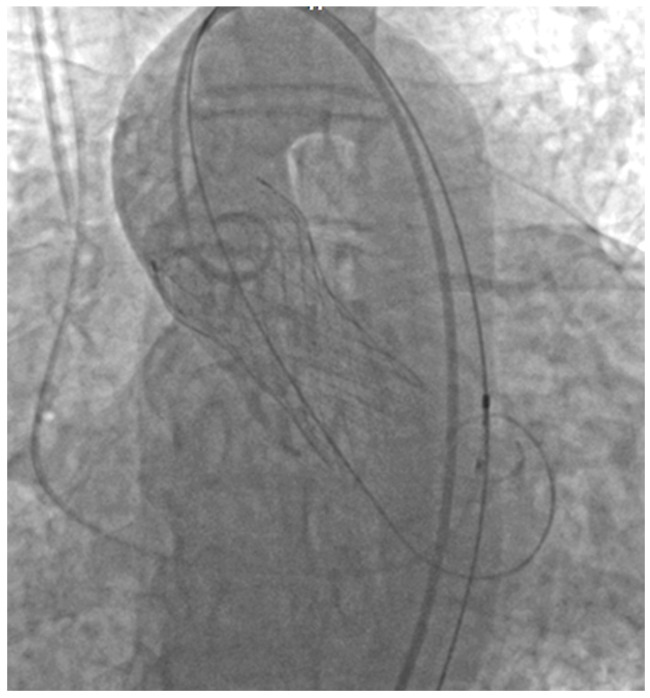
Successful deployment of the aortic valve prosthesis with optimal positioning confirmed. A pigtail catheter is positioned at the aortic valve (AV) to monitor hemodynamic parameters during deployment. An electrostimulation lead is visible in the right ventricle (RV), ensuring temporary pacing support throughout the procedure to allow valve deployment, as well as a prophylactic measure in case of iatrogenic 3rd-degree heart block.

TAVR was successfully performed under deep sedation via a bilateral femoral approach, with implantation of a 29 mm transcatheter aortic valve prosthesis. The procedure had a favorable outcome with no periprocedural complications. Postprocedural echocardiography showed mild aortic regurgitation (Grade I) due to a paravalvular leak, which was hemodynamically insignificant. The patient’s clinical course was favorable, with no periprocedural complications (Figure 7).

At the six-month follow-up, the patient presented improved clinical status, without exertional angina, but with residual dyspnea upon moderate physical exertion. Their biological parameters (coagulation, anemia, and heart failure biomarkers) improved significantly (Table 2). The mean aortic pressure gradient determined via echocardiography remained relatively stable, with a mild paraprosthetic leak, without hemodynamic significance. The fecal immunochemical test was negative.

## 3. Discussion

This is a case of symptomatic aortic stenosis in a 72-year-old female patient with high surgical risk and an indication for valve prosthesis (EuroScore II = 11.36%). The paraclinical analysis revealed severe anemia, which triggered a series of additional investigations that finally led to the patient being diagnosed with Heyde syndrome.

Degenerative aortic stenosis is the most common valvular valve disease encountered in clinical practice, especially in elderly patients [8]. They usually present with multiple comorbidities, resulting in poor functional status and prognosis. In patients with degenerative aortic stenosis, the prevalence of anemic syndrome is much higher than in the general population; the etiology is multifactorial, and it is reported in more than 50% of patients proposed for TAVR [9]. Previous medication (OR = 4.90, 95% CI [3.08–7.80]), male sex (OR = 1.69, 95% CI [1.32–2.16]), history of malignancy (OR = 1.44, 95% CI [1.03–2.09]), and peripheral arterial disease (OR = 1.33, 95% CI [1.04–1.70]) were cited as risk factors for anemia. The presence of anemia (Hb < 10 g/dL) before TAVR did not correlate with 30-day mortality (HR = 1.72, 95% CI [0.96–3.12]; *p* = 0.073), but it correlated with 1-year mortality (HR = 2.78, 95% CI [1.60–4.82]) [9], as well as 3-year mortality, as reported in a meta-analysis [10]. Other studies have reported an increased incidence of anemia in patients with moderate or severe aortic stenosis, which correlates with an increased all-cause mortality in patients before valve prosthesis (HR = 2.26, 95% CI [1.29–3.97], *p* = 0.005) [10].

**Figure 8:**
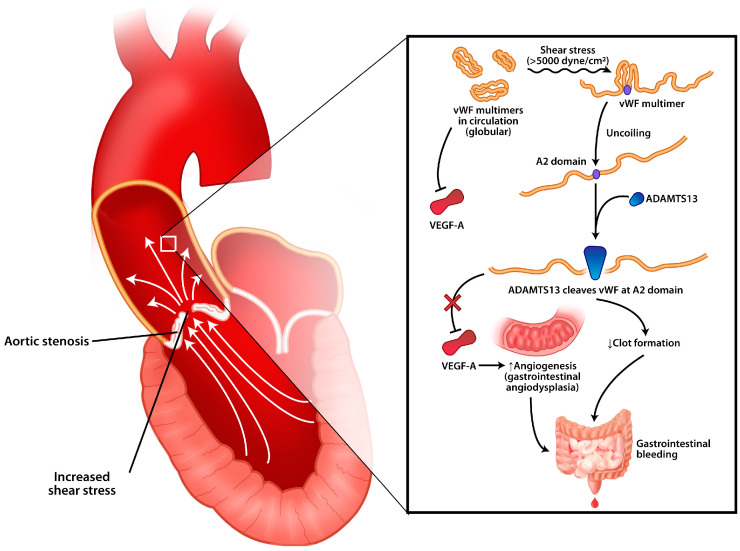
The pathophysiology of Heyde’s syndrome. Severe aortic stenosis produces high shear stress (>5000 dyne/cm^2^: the threshold for von Willebrand factor to change its conformation from globular to elongated shape). The elongated shape of the vWF exposes the A2 domain, allowing ADAMTS13 to bind and cleave vWF multimers into monomers, thereby reducing its clotting activity and VEGF-A inhibition. Clotting activity reduction predisposes the patient to bleeding, especially from the gastrointestinal mucosa. Increased concentrations of VEGF-A promote angiogenesis, leading to gastrointestinal angiodysplasia.

Heyde’s syndrome is characterized by the presence of angiodysplasia-type gastrointestinal bleeding in patients with moderate/severe aortic stenosis [6]. In recent years an acquired von Willebrand factor deficiency has been added to the syndrome definition, a deficiency that occurs through the loss of large multimeric von Willebrand factor multimeric molecules [11]. This is thought to be due to the increased shear stress generated by an intensely calcified aortic valve, which causes a specific change in the von Willebrand factor, namely a decrease in high-molecular-weight vWF multimers [7]. The decrease in the number of multimeric components of the von Wilebrand factor is associated with angiodysplasia appearing in the gastrointestinal mucosa, especially in the ascending portion of the colon, which causes bleeding [12,13] (Figure 8).

But is this the only mechanism? Shear stress is present in intensely calcified medium/severe aortic stenosis, but not all patients present gastrointestinal bleeding.

However, if a patient with degenerative medium/severe aortic stenosis presents gastrointestinal bleeding, they should be fully investigated for a differential diagnosis before the diagnosis of Heyde syndrome. Endoscopy helps in the differential diagnosis, and angiodysplasia of the gastrointestinal mucosa is diagnosed in 80% of cases. In our case, repeated endoscopy did not identify the source of bleeding, even though the patient had an acquired von Willebrand factor deficiency. The bleeding was revealed by video capsule endoscopy (VCE), which is now the gold standard in the diagnosis of gastrointestinal angiodysplasia when endoscopy is negative [14]. Although some cases require endoscopic treatment, most angiodysplasia cases resolve after the correction of aortic stenosis, which was also observed in our case.

Endoscopic treatment for angiodysplasia involves direct coagulation of the lesion by various methods, the most commonly used being argon plasma coagulation (APC) [15,16]. In selected cases, endoscopic clipping or ligation may be necessary to stop bleeding, but these are few, as bleeding is usually diffuse [17].

Some potentially beneficial drugs, along with endoscopic treatment, have been described as therapeutic options for angiodysplasia. Initially, the treatment should be supportive and the goal is to restore anemia by iron supplementation, or even red cell mass transfusion if necessary [18]. Drug treatments include hormonal therapy, somatostatin analogs, and thalidomide [19]. The results regarding hormonal therapy have been inconsistent, with some studies reporting a potential benefit from the combination of levonorgestrel (0.10–0.25 mg) and ethinylestradiol (0.02–0.05 mg) [20,21]. These results have not been confirmed in a prospective, randomized-controlled study.

Thalidomide administration might be beneficial in patients with angiodysplasia, as shown in a number of case reports [22,23,24], due to its effect on inhibiting vascular endothelial growth factor (VEGF)-dependent angiogenesis, thereby suppressing abnormal blood vessel formation in angiodysplasias. However, thalidomide should be administered with caution due to its reported adverse effects, such as neuropathy and sepsis, and it may even worsen the anemia [25].

Somatostatin analogs induce selective vasoconstriction in splanchnic vessels, reducing blood flow by up to 50% in hepatic, splenic, and portal vascular beds via direct action on vascular somatostatin receptors SST2 and SST5, which are overexpressed in the enteric circulation, but without a net benefit on primary bleeding [26,27]. Of the somatostatin analogs, the subcutaneous administration of octreotide had a positive impact on bleeding recurrence, as shown in some case reports and small cohorts of patients (one of the studies also involving patients with von Willebrand factor deficiency [28,29,30,31]). Goldstein et al. showed that although somatostatin analogs (octreotide) have no effect on initial bleeding, their subcutaneous administration greatly reduced the incidence of rebleeding after initial endoscopic treatment [32].

Bevacizumab is a recombinant humanized monoclonal antibody that binds VEGF with subsequent angiogenesis suppression. Primarily used in the treatment of metastatic cancers, bevacizumab has also been employed in gastrointestinal angiodysplasia with refractory hemorrhage [15]. However, further studies are required to investigate the therapeutic potential and safety profile in gastrointestinal angiodysplasia and Heyde syndrome, respectively.

The most effective initial therapy for angiodysplasia was endoscopic treatment, compared to hormonal or somatostatin analogs, but about 1/3 of patients experienced rebleeding after the initial endoscopic approach [32]. Although some cases require endoscopic treatment, some cases of angiodysplasia remit after the correction of aortic stenosis [16,17,33], which was also observed in our case report.

The medical management of acquired von Willebrand factor deficit can either (1) improve the production and release of vWF from endothelial cells or (2) provide products containing vWF to mitigate the deficit.

Desmopressin (1-deamino-8-D-arginine vasopressin) binds to endothelial V2 receptors (Gs protein-coupled receptors), which consequently increases the activity of protein kinase A and cyclic AMP (cAMP) production. Cyclic AMP-dependent cytoskeleton modifications trigger the exocytosis of Weibel–Palade bodies containing vWF in the bloodstream [34].

Von Willebrand factor supplementation can be achieved through the administration of fresh frozen plasma, cryoprecipitate, inactivated vWF/VIII concentrates, and recombinant vWF analogs (Vovendi). Vovendi increases vWF activity substantially, mainly because of its resistance to ADAMTS13-mediated proteolysis. Although they provide a solution to vWF deficiency, one must use them perioperatively with caution given the high thromboembolic risk [35].

## 4. Conclusions

Patients with moderate/severe degenerative aortic stenosis often present with associated anemia, which is correlated with a poor prognosis. Efforts should be made to detect the cause of anemia, including digestive endoscopy. If this is negative, a gastrointestinal cause should not be excluded, and the use of endoscopic capsule endoscopy might be a diagnostic solution. The association between aortic stenosis, anemia, and gastrointestinal angiodysplasia has been termed Heyde syndrome. Endoscopic treatment with argon is the preferred method to control bleeding but is hampered by a high recurrence rate. In some cases, valve prosthesis via classical surgery or TAVR (in patients at high surgical risk) is curative for anemic syndrome.

## Figures and Tables

**Table 1:** Patient’s laboratory findings from day 0 to day 7 of admission. Severe microcytic, hypochromic anemia was observed at admission, with high levels of NT-proBNP, suggestive of cardiac decompensation. The von Willebrand ristocetin-to-antigen ratio was lower than the reference value, suggestive of acquired von Willebrand disease type 2A.

Laboratory Investigation	Admission (Day 0)	Day 3	Day 7	Reference Values
Red blood cells	2.45 × 10^6^/μL	2.98 × 10^6^/μL	3.42 × 10^6^/μL	3.5–5.5 × 10^6^/μL
Hemoglobin	6.8 g/dL	7.9 g/dL	9.6 g/dL	11–16 g/dL
Hematocrit (Ht)	27.2%	31.6%	38.4%	37–47%
Mean corpuscular volume (MCV)	74 fL	78 fL	86 fL	80–100 fL
Mean corpuscular hemoglobin (MCH)	25 pg	26.5 pg	28 pg	27–31 pg
Mean corpuscular hemoglobin concentration (MCHC)	25 g/dL	24.9 g/dL	25 g/dL	32–36 g/dL
Platelet count	178 × 10^3^/L	188 × 10^3^/L	211 × 10^3^/L	100–300 × 10^3^/μL
aPTT	28 s	31 s	35 s	20–50 s
INR	0.98	0.97	1	0.8–1.2
Serum iron	25 μg/dL	73 μg/dL	94 μg/dL	50–170 μg/dL
Ferritin	10 ng/dL	17 ng/dL	28 ng/dL	15–205 ng/dL
NT-proBNP	3176 pg/mL	2655 pg/mL	1035 pg/mL	<50 pg/mL
vWF antigen	-	-	43 IU/L	50–200 IU/L
vWF: Rco	-	-	26.3 IU/L	50–150 IU/L
vWF: Rco/VWF antigen ratio	-	-	0.61	>0.7

**Table 2:** Patient’s laboratory findings at six-month follow-up. The severe microcytic, hypochromic anemia resolved, with a hemoglobin concentration close to the lower limit of normal. NT-proBNP levels dropped significantly, suggesting efficient management of heart failure decompensation and its risk factors. The von Willebrand ristocetin-to-antigen ratio was in the interval of reference values, suggesting that the coagulation disorder resolved after TAVR.

Laboratory Investigation	2-Month Follow-Up	Reference Values
Red blood cells	3.53 × 10^6^/μL	3.5–5.5 × 10^6^/μL
Hemoglobin	9.8 g/dL	11–16 g/dL
Hematocrit (Ht)	39.2%	37–47%
Mean corpuscular volume (MCV)	91 fL	80–100 fL
Mean corpuscular hemoglobin (MCH)	29 pg	27–31 pg
Mean corpuscular hemoglobin concentration (MCHC)	33 g/dL	32–36 g/dL
Platelet count	206 × 10^3^/L	100–300 × 10^3^/μL
aPTT	38 s	20–50 s
INR	0.98	0.8–1.2
NT-proBNP	152 pg/mL	<50 pg/mL
vWF antigen	88	50–200 IU/L
vWF: Rco	73.9	50–150 IU/L
vWF: Rco/VWF antigen ratio	0.84	>0.7

## Data Availability

The original contributions presented in this study are included in the article. Further inquiries can be directed to the corresponding author.

## Abbreviations

The following abbreviations are used in this manuscript:

AV	Aortic valve
ADAMTS13	A disintegrin and metalloproteinase with a thrombospondin type 1 motif, member 13
BMI	Body mass index
BSA	Body surface area
ECG	Electrocardiogram
FIT	Fecal immunochemical test
LV	Left ventricle
LAO	Left anterior oblique
RAO	Right anterior oblique
RCo	Ristocetin cofactor
RV	Right ventricle
TAVR	Transcatheter aortic valve replacement
TEE	Transesophageal echocardiography
TTE	Transthoracic echocardiography
VCE	Video capsule endoscopy
vWF	Von Willebrand factor
